# The efficacy and safety of transcranial direct current stimulation for cerebellar ataxia: a systematic review and meta-analysis

**DOI:** 10.1007/s12311-020-01181-z

**Published:** 2021-02

**Authors:** Tiffany X. Chen, Chen-Ya Yang, Gloria Willson, Chih-Chun Lin, Sheng-Han Kuo

**Affiliations:** 1 Department of Biomedical Engineering, Whiting School of Engineering, Johns Hopkins University, Baltimore, MD, USA.; 2 Department of Neurology, College of Physicians and Surgeons, Columbia University, New York, NY, USA.; 3 Initiative for Columbia Ataxia and Tremor, Columbia University, New York, NY, USA.; 4 Augustus C. Long Health Sciences Library, Columbia University New York, NY, USA.

**Keywords:** transcranial direct current stimulation, electric stimulation, ataxia, cerebellum, gait

## Abstract

**Background –:**

A promising new approach, transcranial direct current stimulation (tDCS) has recently been used as a therapeutic modality for cerebellar ataxia. However, the strength of the conclusions drawn from individual studies in the current literature may be constrained by the small sample size of each trial.

**Methods –:**

Following a systematic literature retrieval of studies, meta-analyses were conducted by pooling the standardized mean differences (SMDs) using random-effects models to assess the efficacy of tDCS on cerebellar ataxia, measured by standard clinical rating scales. Domain-specific effects of tDCS on gait and hand function were further evaluated based on 8-meter walk and 9-hole peg test performance times, respectively. To determine the safety of tDCS, the incidences of adverse effects were analyzed using risk differences.

**Results –:**

Out of 293 citations, 5 randomized controlled trials involving a total of 72 participants with cerebellar ataxia were included. Meta-analysis indicated a 26.1% (*p* = 0.003) improvement in ataxia immediately after tDCS with sustained efficacy over months (28.2% improvement after 3 months, *p* = 0.04) when compared to sham stimulation. tDCS seems to be domain-specific as the current analysis suggested a positive effect on gait (16.3% improvement, *p* = 0.04), however failed to reveal differences for hand function (*p* = 0.10) with respect to sham. The incidence of adverse events in tDCS and sham groups was similar.

**Conclusion –:**

tDCS is an effective intervention for mitigating ataxia symptoms with lasting results that can be sustained for months. This treatment shows preferential effects on gait ataxia and is relatively safe.

## 1.0 Introduction

Cerebellar ataxia arises from a variety of genetic or acquired etiologies that ultimately lead to cerebellar dysfunction. As a result, patients with cerebellar ataxia may suffer from eye movement abnormalities, limb incoordination, gait instability, and speech impairment. The available treatment options for cerebellar ataxia are rather limited, leaving many patients with difficulty accomplishing activities of daily living.

Purkinje cells are the principal neurons in the cerebellar cortex and have very high intrinsic excitability [1]. The firing patterns of Purkinje cells are also intricately modulated by excitatory inputs, including climbing fibers and parallel fibers [2]. Cerebellar ataxia is often the result of pathologic processes that cause Purkinje cell firing patterns to become slow and irregular [3]. Therefore, neuromodulation to improve Purkinje cell physiology may provide symptomatic benefits in patients with cerebellar ataxia.

In contrast to many other neuromodulatory methods, transcranial direct current stimulation (tDCS) is a non-invasive technique that utilizes a low-voltage current for polarity-dependent manipulation of cortical excitability to promote neuroplasticity in targeted brain regions [4]. The stimulated areas are generally assumed to be localized under the electrode, allowing the cerebellar cortex to be a potential target for neuromodulation by tDCS. With its low cost and ease of execution, tDCS is also particularly suitable for future large-scale clinical trials or home-based therapies.

Based on these unique features, tDCS was first proposed as a neuromodulatory intervention for cerebellar ataxia by Manto et al. in 2008 [5]. Several clinical trials were later conducted using techniques outlined by Ferrucci et al. to investigate the utility of this therapy, though the validity of each study alone may be limited by the small sample sizes (*n* = 1 – 20) of individual trials [6–11]. Therefore, it is difficult to make definitive conclusions regarding tDCS for cerebellar ataxia. Our goals are to determine the (1) overall efficacy of tDCS for cerebellar ataxia, (2) therapeutic window of tDCS, (3) potential domain-specificity (i.e. hand function vs. gait) of tDCS, and (4) safety of tDCS in individuals with cerebellar ataxia.

## 2.0 Methods

### 2.1 Study Selection

This systematic review and meta-analysis adhered to the Preferred Reporting Items for Systematic Reviews and Meta-Analyses (PRISMA) and was registered with PROSPERO (ID: CRD42020151760). The study did not require ethics committee approval as all data were fully anonymized and there were no primary data collection. PUBMED, EMBASE, Cochrane Central Register of Controlled Trials (CENTRAL), and WEB OF SCIENCE were searched. The search strategy included keywords relating to or describing the intervention - *transcranial direct current stimulation, tDCS, brain stimulation, cerebellar ataxia, spinocerebellar ataxia* (Supplementary Table 1). English-only and publication date filters to include studies published between January 2005 to October 11, 2019 (date searches were conducted) were applied to the search strategies. Citations from all databases were uploaded to COVIDENCE and the result set was de-duplicated.

All items found in the literature during the identification phase were screened by at least 2 authors (TXC and CYY) who examined titles and abstracts for the following inclusion criterion: randomized sham-controlled trials (RCTs) to assess either or both the efficacy and safety of tDCS in cerebellar ataxia. During the full-text review, at least 2 authors (TXC and CYY) independently assessed each study and came to a consensus for inclusion based on the following predetermined criteria: studies needed to either (1) measure ataxia using standard clinical rating scales or (2) report adverse effects. Poster abstracts and duplicates were excluded. Any disagreements between the 2 authors performing screening and exclusion were resolved through discussion with a third author (SHK).

### 2.2 Data Extraction

The following data were extracted from the identified publications: (1) study design (sample size, blinding, controls, intervention), (2) participant characteristics (age, sex, diagnosis), (3) tDCS protocol (current polarity, current intensity, electrode montage, number of sessions, duration), (4) outcome measures evaluating motor function (scale for the assessment and rating of ataxia (SARA), international cooperative ataxia rating scale (ICARS), 8-meter walk test, 9-hole peg test), and (5) report of adverse effects (frequency of symptoms).

### 2.3 Statistical Analysis

To determine the efficacy of tDCS, primary analysis of the pooled standardized mean differences (SMDs) calculated from the change in ataxia rating scores from baseline at various post-tDCS assessment timepoints based on the Cochrane Handbook for Systematic Reviews of Interventions Version 5.1.0 was performed [12]. Secondary analysis of the pooled SMDs computed from the change in performance time on the 8-meter walk test and 9-hole peg test from baseline at different post-tDCS timepoints to assess gait ataxia and hand ataxia, respectively. Using the Cochrane Review Manager 5.3, forest plots were created by pooling SMDs using a random-effects model with 95% confidence intervals according to inverse-variance weighting. A random-effects model was chosen to allow for treatment effects to vary across studies [13]. Practical interpretation of the determined effect size was enhanced by calculating Cohen’s U3, an index used to determine the percentile change in an intervention group compared to a control group [14]. To evaluate the safety of tDCS, the risk difference of reported adverse effects was determined and pooled using a random-effects model with 95% confidence intervals according to Mantel-Haenszel weighting. Heterogeneity was quantified by calculating the I^2^ statistic between studies and results were considered significant if the *p*-value was < 0.05. Since fewer than 10 studies were eventually included, funnel plots were not created [15].

### 2.4 Quality Assessment

To rate the scientific rigor of individual studies, the Cochrane Risk of Bias (RoB2) tool for RCTs was used. In this study, two authors (TXC and GW) independently rated each RCT using this tool, and then all authors came to a consensus on each domain.

## 3.0 Results

### 3.1 Study Inclusion

**Fig.1:**
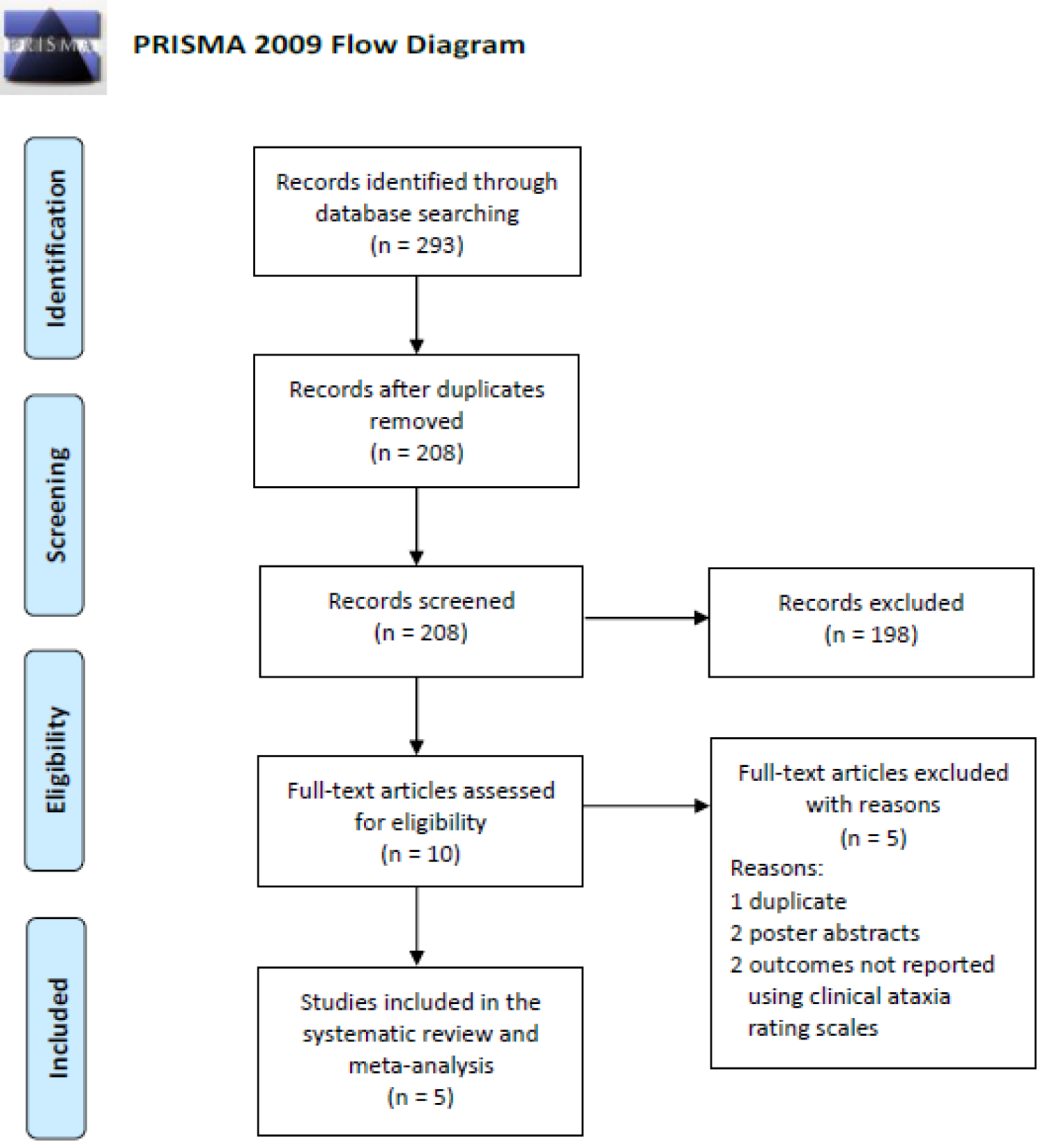
Preferred Reporting Items for Systematic Reviews and Meta-Analyses (PRISMA) study selection flow diagram

We identified 293 citations from all databases. After duplicates were removed, 208 titles and abstracts were filtered for relevance, resulting in the subsequent exclusion of 198 references. The remaining 10 articles were evaluated in full text and 5 articles were excluded since they were either duplicates with data already included, poster abstracts with insufficient data presented, or studies that did not use clinical ataxia rating scales as outcome measures. The final review included 5 RCTs involving a total of 72 participants with cerebellar ataxia (Figure 1) [10,16–19]. Of these subjects, 45.83% have hereditary ataxia, 36.11% have idiopathic ataxia, 8.33% have ataxic cerebral palsy, and 9.72% have ataxia of unreported etiology. Across studies, the mean sample size was 14 subjects (range of 6–20) and the mean participant age was 40.94 years (range of 5–72 years). One study involved gait training as a concurrent intervention [19]. The characteristics of each study and a description of the participants and tDCS protocols are further summarized in Table 1 and Supplementary Table 2 respectively.

Of the 5 RCTs [10,16–19] included in this study, 4 RCTs [10,16–18] measured ataxia with clinical rating scales and were thus eligible for meta-analysis examining tDCS efficacy. The meta-analysis assessing tDCS safety included all 3 RCTs [10,17,19] that reported on the participant incidence of adverse effects in both active and control groups. From the risk of bias assessment, all included RCTs were determined to be of high quality with only a few concerns (Supplementary Figure 1) [10,16–19].

### 3.2 Efficacy of tDCS

**Fig.2:**
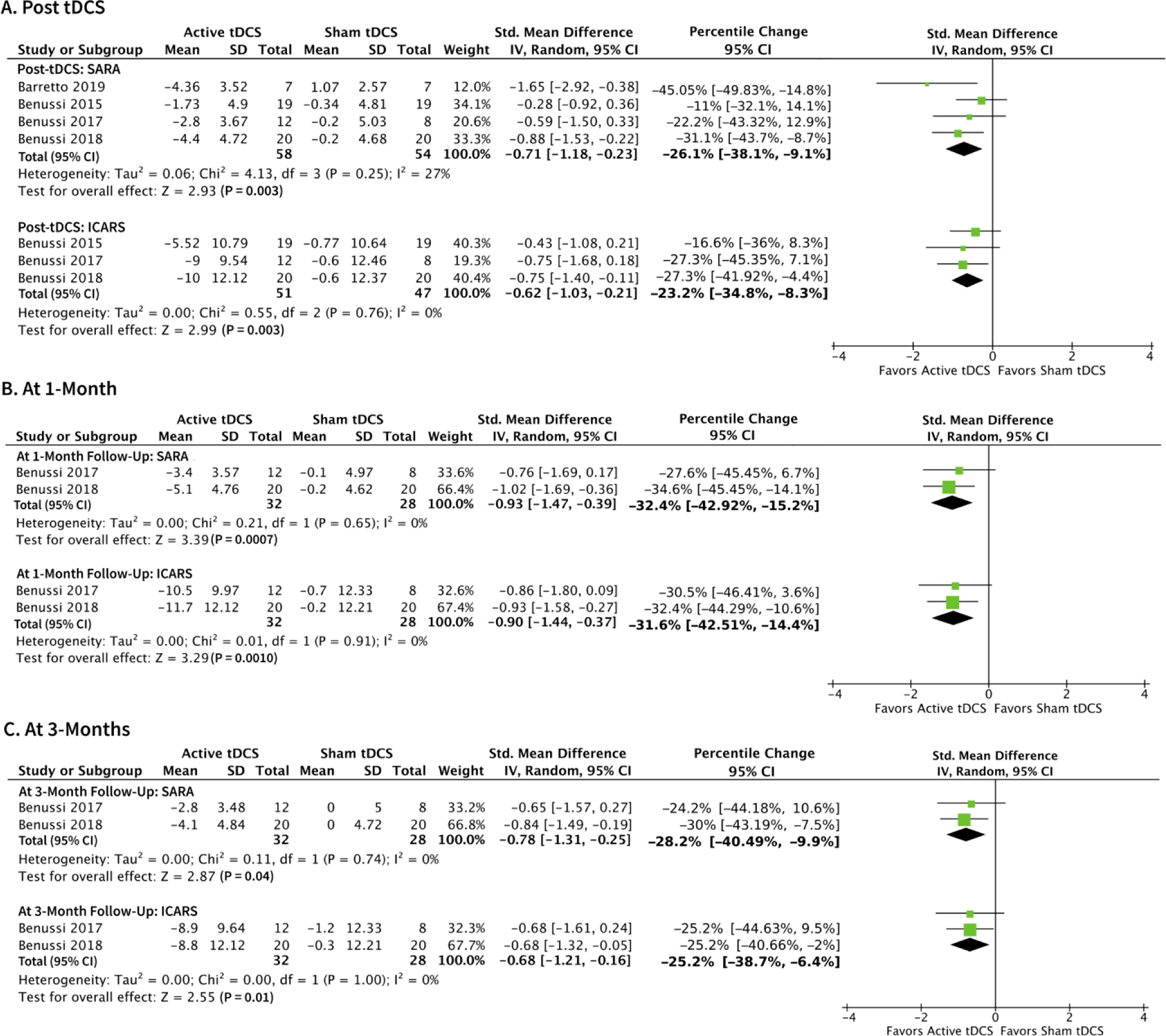
Forest plots of the meta-analysis on the effects of tDCS on ataxia as determined by standard clinical rating scales (**A**) initially following the intervention, (**B**) 1 month following the intervention, and (**C**) 3 months following the intervention. Standardized mean differences were calculated from the changes in ataxia rating scale scores from baseline and pooled using inverse-variance weighted random effects models with the overall total effect sizes indicated by diamonds. Abbreviations: SARA, scale for assessment and rating of ataxia; ICARS, international cooperative ataxia rating scale

Primary analysis of tDCS efficacy using SARA score changes showed that the improvement in ataxia severity from baseline to the initial post-intervention assessment is 26.1% (95% CI = 9.1% to 38.1%, 4 RCTs [10,16–18], *p* = 0.003; *I*^*2*^ = 27%) better than the sham-stimulation (Figure 2A). These effects were sustained throughout all follow-ups, with ataxia improvement of 32.4% (95% CI = 15.2% to 42.92%, 2 RCTs [10,17], *p* = 0.0007; *I*^*2*^ = 0%) at 1 month post-tDCS and 28.2% (95% CI = 9.9% to 40.49%, 2 RCTs [10,17], *p* = 0.04; *I*^*2*^ = 0%) at 3 months post-tDCS over the control group (Figure 2B & 2C). Similar benefits were seen in the meta-analysis of changes in ICARS score before and after tDCS. Spanning from immediately to 3 months following tDCS, statistically significant reductions in ataxia, measured by ICARS change from baseline, were seen at all assessment timepoints: 23.2% improvement immediately post-tDCS (95% CI = 8.3% to 34.8%, 3 RCTs [10,17,18], *p* = 0.003; *I*^*2*^ = 0%), 31.6% at 1 month post-tDCS (95% CI = 14.4% to 42.51%, 2 RCTs [10,17], *p* = 0.0010; *I*^*2*^ = 0%), and 25.2% at 3 months post-tDCS (95% CI = 6.4% to 38.7%, 2 RCTs [10,17], *p* = 0.01; *I*^*2*^ = 0%) with respect to sham-control (Figure 2A–2C). One of the studies stimulated at the motor cortex [16], whereas the remaining RCTs targeted the cerebellum [10,17,18]. When excluding the sole study involving motor cortex stimulation [16], improvement after tDCS as measured by SARA score changes from baseline to initial assessment remained comparable and significant (21.6% improvement over sham-stimulation, 95% CI = 6.7% to 33.6%, 3 RCTs [10,17,18], *p* = 0.006, *I*^*2*^ = 0%).

**Fig.3:**
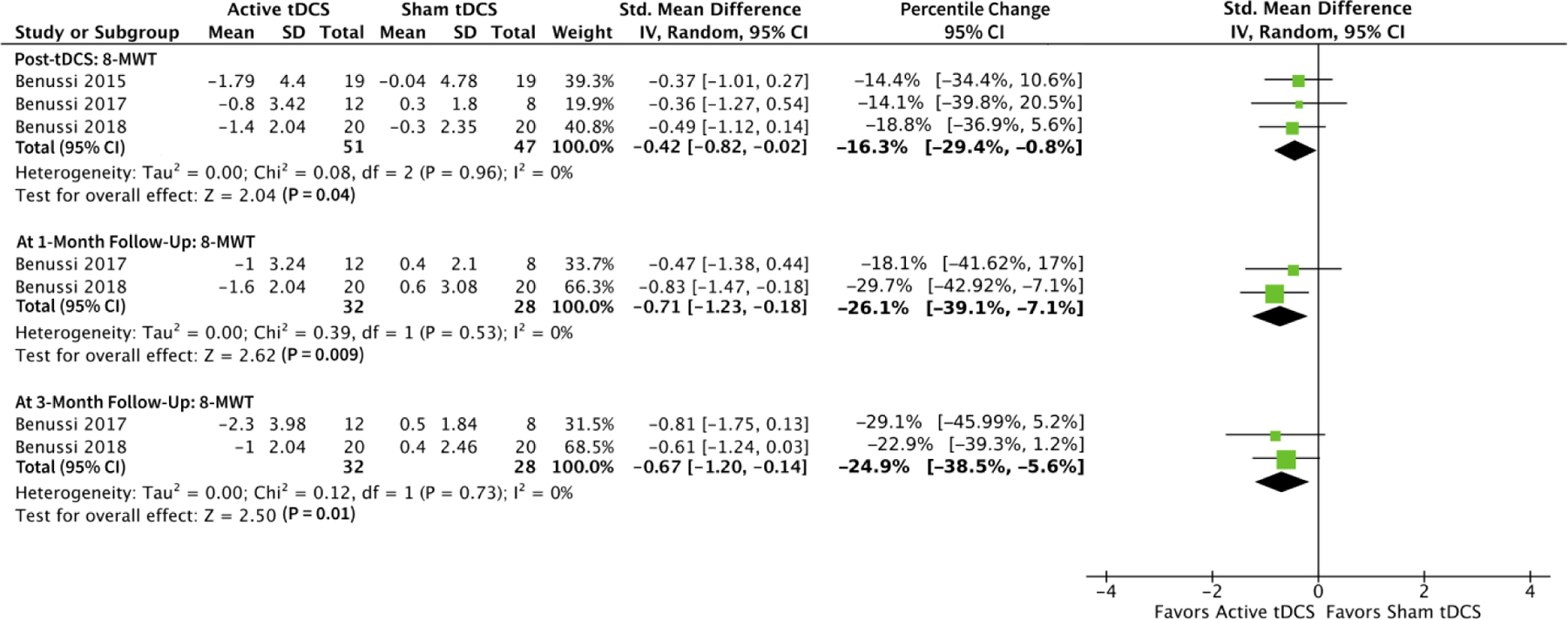
Forest plots of the meta-analysis on the effects of tDCS on 8-meter walk performance post-intervention, 1 month following the intervention, and 3 months following the intervention. Standardized mean differences were calculated from the changes in 8-meter walk times from baseline and pooled using inverse-variance weighted random effects models with the overall total effect sizes indicated by diamonds. Abbreviation: 8-MWT, 8-meter walk time

**Fig.4:**
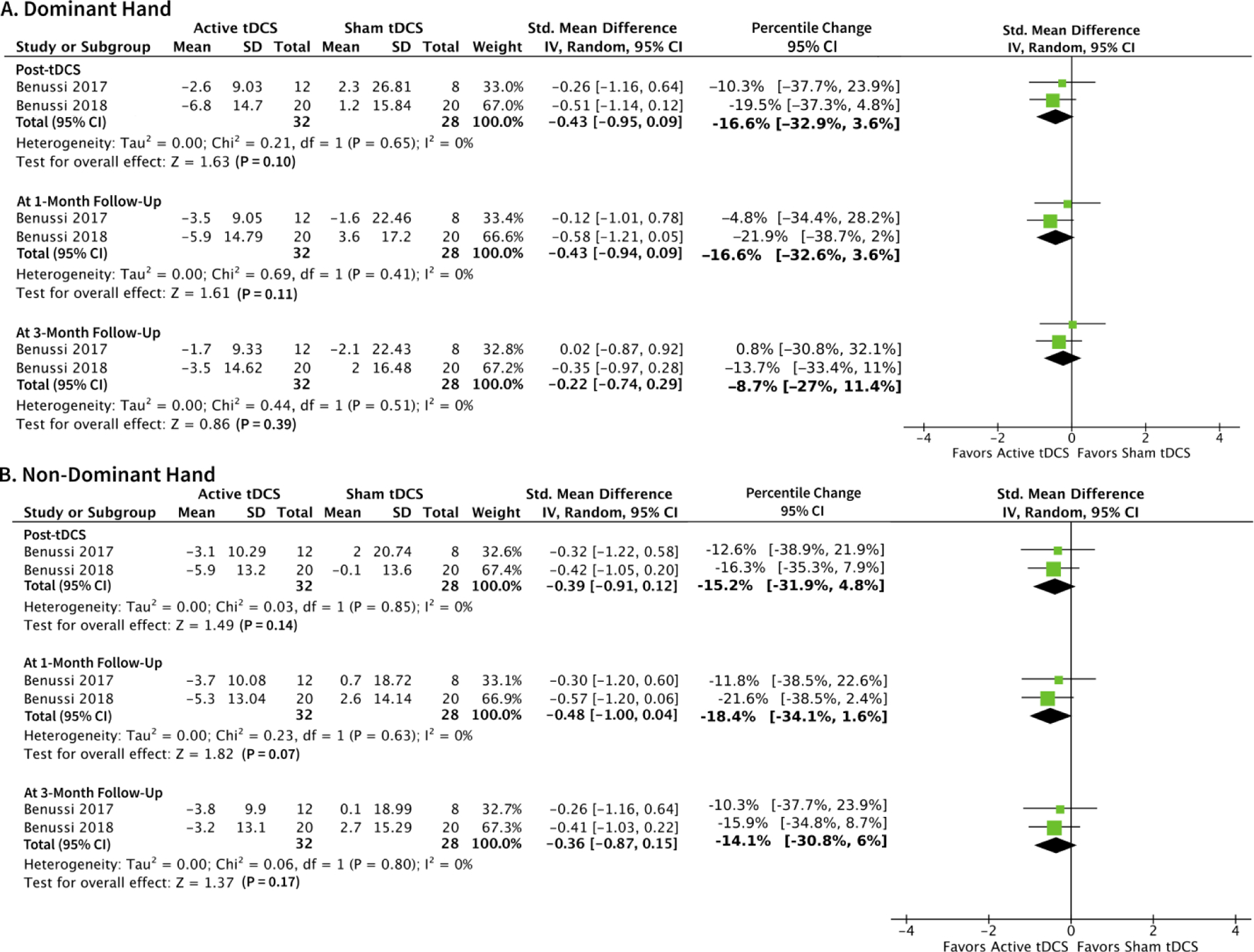
Forest plots of the meta-analysis on the effects of tDCS in 9-peg hole test performance post-intervention, 1 month following the intervention, and 3 months following the intervention for the (**A**) dominant hand and the (**B**) non-dominant hand. Standardized mean differences were calculated from the changes in 9-hole peg test completion times from baseline and pooled using inverse-variance weighted random-effects models with the overall total effect sizes indicated by diamonds

In order to identify potential domain-specific effects, changes in gait and hand function were next investigated and compared. The findings showed that tDCS enhanced gait, as measured by 8-meter walk times, with a 16.3% improvement immediately after stimulation (95% CI = 0.8% to 29.4%, 3 RCTs [10,17,18], *p* = 0.04; *I*^*2*^ = 0%), a 26.1% improvement at 1-month follow up (95% CI = 7.1% to 39.1%, 2 RCTs [10,17], *p* = 0.009; *I*^*2*^ = 0%), and a 24.9% improvement at 3-month follow up (95% CI = 5.6% to 38.5%, 2 RCTs [10,17], *p* = 0.01; *I*^*2*^ = 0%) when compared to sham-stimulation (Figure 3). In contrast, tDCS did not significantly alter hand function, as measured by 9-peg hole test performance, with no statistically detectable improvement immediately after stimulation (95% CI = −3.6% to 32.9%, 2 RCTs [10,17], *p* = 0.10 in the dominant hand and 95% CI = −4.8% to 31.9%, 2 RCTs [10,17], *p* = 0.14 in the non-dominant hand), at 1-month follow up (95% CI = −3.6% to 32.6%, 2 RCTs [10,17], *p* = 0.11 in the dominant hand and 95% CI = −1.6% to 34.1%, 2 RCTs[10,17], *p* = 0.07 in the non-dominant hand), and at 3-month follow up (95% CI = −11.4% to 27%, 2 RCTs [10,17], and *p* = 0.39 in the dominant hand, and 95% CI = −6% to 30.8%, 2 RCTs [10,17], *p* = 0.17 in the non-dominant hand) with respect to sham-stimulation (Figure 4). Consequently, the difference in the effects on gait and hand ataxia across all timepoints suggested the presence of domain-specific efficacy in tDCS for ataxia.

### 3.3 Safety of tDCS

**Fig.5:**
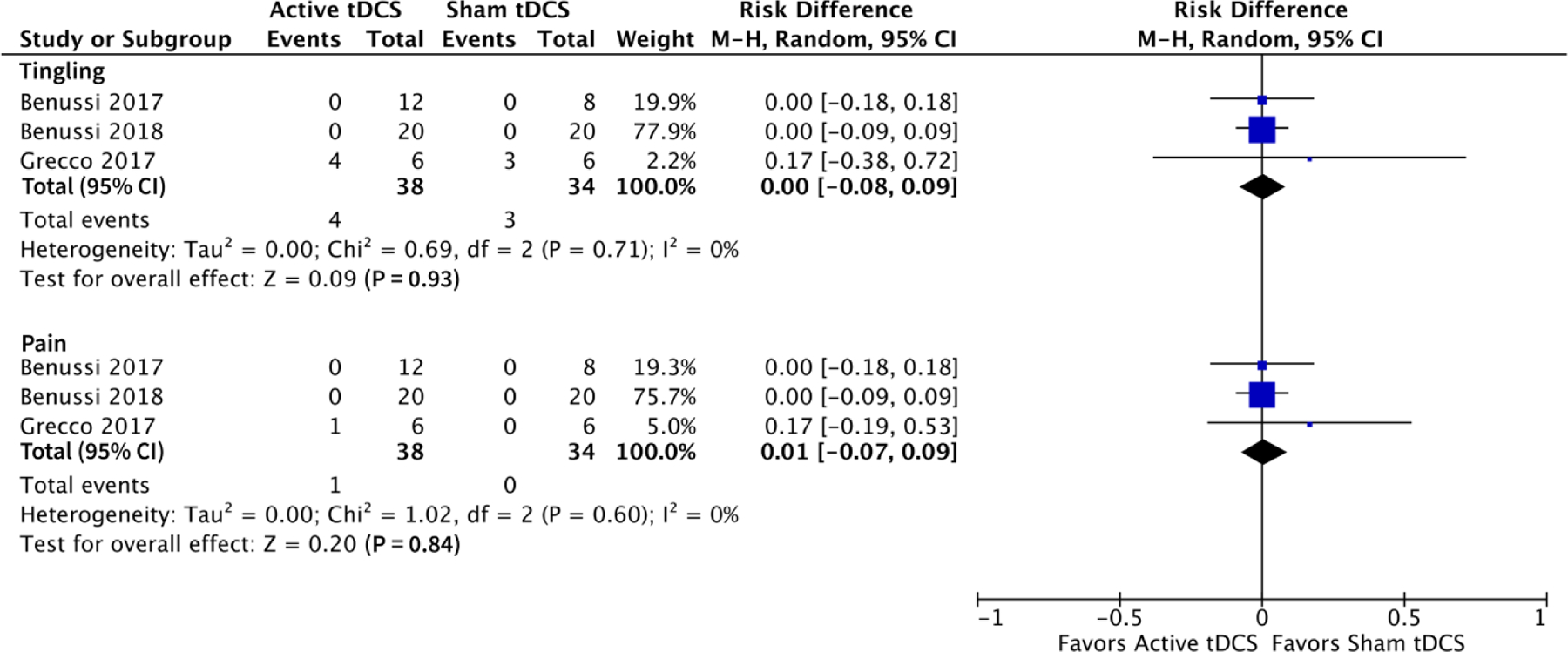
Forest plots of the meta-analysis on the frequency of the adverse effects reported during active-tDCS and sham-tDCS. Risk differences were calculated from the participant incidence of the reported adverse effects and pooled using Mantel-Haenszel weighted random-effects models with overall total effect sizes indicated by diamonds

Across RCTs included in the safety meta-analysis, no severe adverse effects were reported [10,17,19]. Mild side effects were observed with the symptoms being tingling (33%) and pain (33%).^17^ However, the pooled risk differences determined from the participant incidences of the reported symptoms showed no significant difference in risk between the active and sham stimulation groups (risk difference = 0.00, 95% CI = −0.08 to 0.09, 3 RCTs [10,17,19], *p* = 0.93, *I*^*2*^ = 0% for tingling; risk difference = 0.01, 95% CI = −0.07 to 0.09, 3 RCTs [10,17,19], *p* = 0.84, *I*^*2*^ = 0% for pain) (Figure 5).

## 4.0 Discussion

Our study demonstrates the efficacy of tDCS in the treatment of cerebellar ataxia with a lasting impact on patient outcomes extending for months beyond the stimulation regimen. Analysis of current evidence suggests that the effect also appears to be domain-specific given that tDCS provides a greater improvement in gait ataxia when compared to hand ataxia. Finally, we found that tDCS is a relatively safe procedure not associated with any severe adverse events. In summary, tDCS has shown encouraging potential for clinical application in treating patients with ataxia.

The detailed mechanism by which tDCS exerts its effects on cerebellar ataxia requires further investigation. While the paradigm for stimulation and the magnitude of electrical current vary across studies (Supplementary Table 2), enhancing the cerebello-thalamo-cortical loop consistently plays a key role [20]. Since the firing of Purkinje cells often becomes slow and irregular in cerebellar ataxia [21–23], intervention with tDCS potentially enhances Purkinje cell physiology and thus normalizes the dysfunctional cerebellar network. Although electrical modulation of Purkinje cell activity within the cerebello-thalamo-cortical loop is believed to initiate these improvements, tDCS-induced neurochemical changes may intervene to provide lasting effects [24]. It has previously been shown that concentrations of cerebellar neurotransmitters, such as myo-inositol, gamma aminobutyric acid (GABA), and glutamate, are locally altered in response to cerebral tDCS [25,26]. These neurochemical modifications could partly explain the long-term impact of tDCS. While the precise neural mechanism remains to be determined, the effects of tDCS can persist on the order of months, supporting the notion that cerebellar learning can be part of a therapeutic strategy even for cerebellar diseases.

tDCS can be a promising option for the treatment of cerebellar ataxia, however, several important questions remain. First, the exact mechanism of tDCS and the optimal stimulation paradigm need to be established. Studies in animal models will provide mechanistic insights and also help inform new protocols [27]. Second, cerebellar ataxia can be the result of a number of conditions which may preferentially damage different parts of cerebellar circuitry and thus produce variable responses to tDCS. For example, spinocerebellar ataxia type 3 (SCA3) patients have relatively preserved Purkinje cells whereas SCA1, 2, and 6 patients have prominent Purkinje cell loss [28]. Therefore, future studies might explore the heterogeneity between different genotypes of ataxias in response to tDCS or focus on a subgroup of ataxia patients with defined genotypes. Third, the clinical responses to tDCS might differ across various stages of neurodegeneration, which might call for stage-specific stimulation paradigms. Personalization of tDCS protocols may be achieved through computational modelling that effectively allows for individualized dose-control of the applied stimulation intensity [29,30]. Physiological measures such as cerebellar brain inhibition by transcranial magnetic stimulation [31] or cerebello-cortical connectivity by functional magnetic resonance imaging may also be important. A recently developed technique of electroencephalogram over the cerebellar region could potentially serve as an additional physiological measure of target engagement for tDCS protocol development [32]. Furthermore, cerebellar transcranial alternating current stimulation (tACS) allows for frequency-dependent activation of distinct cerebellar networks to drive motor cortex excitability [33], but whether tACS is superior to tDCS remains an open question. Finally, we found that tDCS preferentially improves gait function in patients with cerebellar ataxia based on our analysis of the available literature. Other domains of ataxia such as speech and swallowing function in response to tDCS require further study.

In addition to tDCS, other means of non-invasive cerebellar stimulation have demonstrated promising effects on ataxia. Specifically, applying transcranial magnetic stimulation (TMS) to the cerebellum has been shown to mitigate symptoms of ataxia in patients with SCA [34], cerebellar strokes [35], and idiopathic late onset cerebellar atrophy [36]. Furthermore, cerebellar TMS can have long-range modulatory effects on the cerebral cortex, as evidenced by gait and balance improvements in patients with strokes in the middle cerebral artery territory [37]. Future studies directly comparing the effects of tDCS and TMS will help elucidate the role of these non-invasive neuromodulatory interventions as a part of the treatment algorithm for cerebellar ataxia.

One limitation of this study is that there are few RCTs on the effects of tDCS for ataxic patients in the current literature. Consequently, subgroup analysis of different stimulation parameters and ataxia subtypes could not be performed. Thus, our review also highlights the need for studies targeting specific subgroups of patients and a standard protocol for administering tDCS. Specifically, tDCS target sites should be further investigated considering that both motor cortex and cerebellar stimulation appear to have positive effects on ataxic patients. Our review suggests that more RCTs involving motor cortex stimulation for cerebellar ataxia is especially required to help establish the optimal electrode montage.

Despite the positive findings on tDCS for cerebellar ataxia, its implementation in the clinic remains a challenge. Currently, the majority of neurologists and movement disorders specialists are not familiar with non-invasive neurostimulation techniques. There is also a lack of commercially available tDCS devices that have been shown to be effective for treating cerebellar ataxia. Finally, tDCS for ataxia has yet to be tested in daily clinical practice. Along with further assessment of patient-reported outcomes, the results of these tests will help to provide a better understanding of the impact of tDCS on an ataxic patient’s quality of life. Future efforts on the implementation of tDCS in the ataxia clinic are much needed.

In conclusion, our systematic review and meta-analysis revealed that tDCS could improve symptoms of cerebellar ataxia. Neuromodulation of the cerebellum is an emerging field with widespread implications for the treatment of cerebellar ataxia as well as other neurological disorders [38].

## Supplementary Material

12311_2020_1181_MOESM1_ESM **Supplementary Table 1.** Search strategy used in the review and meta-analysis

12311_2020_1181_MOESM2_ESM **Supplementary Table 2.** tDCS protocols of included studies

12311_2020_1181_MOESM3_ESM **Supplementary Fig.1** Assessment of the quality of included randomized controlled trials with the Cochrane Risk of Bias (RoB2) tool. The following domains of potential bias were considered: (D1) randomization process, (D2) deviations from the intended interventions, (D3) missing outcome data, (D4) measurement of the outcome, and (D5) selection of the reported result

## Figures and Tables

**Table 1.** Characteristics of included studies

Study	Design	Sample Size	Diagnosis	Age (Mean ± SD)	Gender	Motor Outcome Measures	Assessment Time(s)
Barretto et al (2019)^16^	Randomized, double-blind, crossover, sham-tDCS controlled	7	4 Slowly Progressive 3 Non-Progressive	36.6 ± 17.2	3 Males, 4 Females	SARA, Stabilometric Parameters	Pre-intervention, Post-intervention
Benussi et al (2015)^18^	Randomized, doubleblind, crossover, sham-controlled	19	6 MSA-C 5 SCA2 2 SAOA 2 SCA38 1 AOA2 1 FA 1 FXTAS 1 SCA1	53.8 ± 18.4	8 Males, 11 Females	SARA, ICARS, 9HPT, 8MWT	Pre-intervention, Post-intervention
Benussi et al (2017)^17^	Randomized, doubleblind, sham-tDCS controlled	20	5 SAOA 5 SCA2 4 MSA-C 2 SCA38 1 AOA2 1 FA 1 FXTAS 1 SCA14	52.5 ± 17.5	10 Males, 10 Females	SARA, ICARS, 9HPT, 8MWT	Pre-intervention, Post-intervention, 1-Month Follow-Up, 3-Month Follow-Up
Benussi et al (2018)^10^	Randomized, doubleblind, crossover, sham-tDCS controlled	20	7 SCA2 5 MSA-C 4 SAOA 1 AOA2 1 FA 1 SCA14 1 SCA38	54.6 ± 14.5	10 Males, 10 Females	SARA, ICARS, 9HPT, 8MWT	Pre-intervention, Post-intervention, 1-Month Follow-Up, 3-Month Follow-Up
Grecco et al (2017)^19^	Randomized, singleblind, crossover, sham-tDCS + gait training controlled	6	6 Ataxic CP	7.2 ± 2.1	3 Males, 3 Females	PBS, TUG, PEDI, Stabilometric Parameters	Pre-intervention, Post-intervention, 1-Month Follow-Up, 3-Month Follow-Up

**Abbreviations: AOA**, ataxia with oculomotor apraxia; **CP**, cerebral palsy; **FA**, Friedrich’s ataxia; **FXTAS**, fragile-X associated ataxia syndrome; **ICARS**, international cooperative ataxia rating scale; **MSA-C**, cerebellar variant of multiple system atrophy; **PBS**, pediatric balance scale; **PEDI**, pediatric evaluation of disability inventory; **SAOA**, sporadic adult-onset ataxia; **SARA**, scale for assessment and rating of ataxia; **SCA**, spinocerebellar ataxia; **TUG**, timed up and go test; **8MWT**, 8-meter walk time; **9HPT**, 9-hole peg test.

## Abbreviations and Acronyms

ICARS: international cooperative ataxia rating scale
SARA: scale for the assessment and rating of ataxia
SCA: spinocerebellar ataxia
SMD: standardized mean difference
RCT: randomized controlled trial
tACS: transcranial alternating current stimulation
tDCS: transcranial direct current stimulation
TMS: transcranial magnetic stimulation
95% CI: 95% confidence interval
