# Supplementary material for: The efficacy and safety of transcranial direct current stimulation for cerebellar ataxia: a systematic review and meta-analysis
Source: Cerebellum. Author manuscript; Available in PMC 2022 Feb 1. (PMC7864859; doi:10.1007/s12311-020-01181-z)
Supplement: 12311_2020_1181_MOESM1_ESM — Supplementary Table 1. Search strategy used in the review and meta-analysis [file NIHMS1623197-supplement-12311_2020_1181_MOESM1_ESM.doc]

**Supplementary Table 1. Search strategy used in the review and meta-analysis.**

| **Database** | **Number of Results** | **Search Strategy** |
| --- | --- | --- |
| PubMed | 59 | ("transcranial direct current stimulation"[MeSH Terms] OR ("transcranial"[All Fields] AND "direct"[All Fields] AND "current"[All Fields] AND "stimulation"[All Fields]) OR "transcranial direct current stimulation"[All Fields] OR "tdcs"[All Fields] OR "brain stimulation"[All Fields]) AND ("therapy"[Subheading] OR "therapy"[All Fields] OR "treatment"[All Fields] OR "therapeutics"[MeSH Terms] OR "therapeutics"[All Fields]) AND ("cerebellar ataxia"[MeSH Terms] OR ("cerebellar"[All Fields] AND "ataxia"[All Fields]) OR "cerebellar ataxia"[All Fields] OR "spinocerebellar ataxia"[All Fields]) [English]/lim |
| Embase | 124 | ('transcranial' AND 'direct' AND 'current' AND 'stimulation' OR 'transcranial direct current stimulation' OR 'tdcs' OR 'brain stimulation') AND ('therapy' OR 'treatment' OR 'therapeutics') AND ('cerebellar' AND 'ataxia' OR 'cerebellar ataxia' OR 'spinocerebellar ataxia') AND [English]/lim |
| Cochrane Library (Cochrane Central Register of Controlled Trials) | 22 | ('transcranial' AND 'direct' AND 'current' AND 'stimulation' OR 'transcranial direct current stimulation' OR 'tdcs' OR 'brain stimulation') AND ('therapy' OR 'treatment' OR 'therapeutics') AND ('cerebellar' AND 'ataxia' OR 'cerebellar ataxia' OR 'spinocerebellar ataxia') [English]/lim |
| Web of Science | 88 | ('transcranial' AND 'direct' AND 'current' AND 'stimulation' OR 'transcranial direct current stimulation' OR 'tdcs' OR 'brain stimulation') AND ('therapy' OR 'treatment' OR 'therapeutics') AND ('cerebellar' AND 'ataxia' OR 'cerebellar ataxia' OR 'spinocerebellar ataxia') [English]/lim |
