# Supplementary material for: The efficacy and safety of transcranial direct current stimulation for cerebellar ataxia: a systematic review and meta-analysis
Source: Cerebellum. Author manuscript; Available in PMC 2022 Feb 1. (PMC7864859; doi:10.1007/s12311-020-01181-z)
Supplement: 12311_2020_1181_MOESM2_ESM — Supplementary Table 2. tDCS protocols of included studies [file NIHMS1623197-supplement-12311_2020_1181_MOESM2_ESM.doc]

**Supplementary Table 2.** tDCS protocols of included studies.

| **Study** | **Concurrent Intervention(s)** | **Number of Sessions** | **Stimulation Polarity** | **Target Electrode(s) Placement** | **Reference Electrode Placement** | **Current Intensity and Duration** | **Electrode Size** |
| --- | --- | --- | --- | --- | --- | --- | --- |
| Barretto et al  (2019)^16^ | None reported | 5 (Across 5 days) | Anode | M1 (left M1 followed by right M1) | Contralateral supraorbital region | 2 mA, 40 min (20 min/side of motor cortex) | 5 x 7 cm^2^ |
| Benussi et al (2015)^18^ | None reported | 1 | Anode | Cerebellum | Right deltoid muscle | 2 mA, 20 min | 7 x 5 cm^2^ |
| Benussi et al (2017)^17^ | None reported | 10 (5 days/week for 2 weeks) | Anode | Cerebellum (2 cm under inion) | Right deltoid muscle | 2 mA, 20 min | 7 x 5 cm^2^ |
| Benussi et al (2018)^10^ | None reported | 10 (5 days/week for 2weeks) | Anode and cathode | Cerebellum (anode 2 cm under inion),  Spine (cathode 2 cm under T11) | Not applicable^†^ | 2 mA, 20 min | 7 x 5 cm^2^ (anode),  8 x 6 cm^2^ (cathode) |
| Grecco et al (2017)^19^ | Gait training | 5 (Across 2 weeks) | Anode | Cerebellum (anode 1 cm under inion), Central supraorbital region (cathode) | Not applicable^†^ | 1 mA, 20 min | 5 x 7 cm^2^ |

^†^Both the anodal and cathodal electrodes are considered to be active in this montage.
