# Supplementary figures and images for: The efficacy and safety of transcranial direct current stimulation for cerebellar ataxia: a systematic review and meta-analysis
Source: Cerebellum. Author manuscript; Available in PMC 2022 Feb 1. (PMC7864859; doi:10.1007/s12311-020-01181-z)

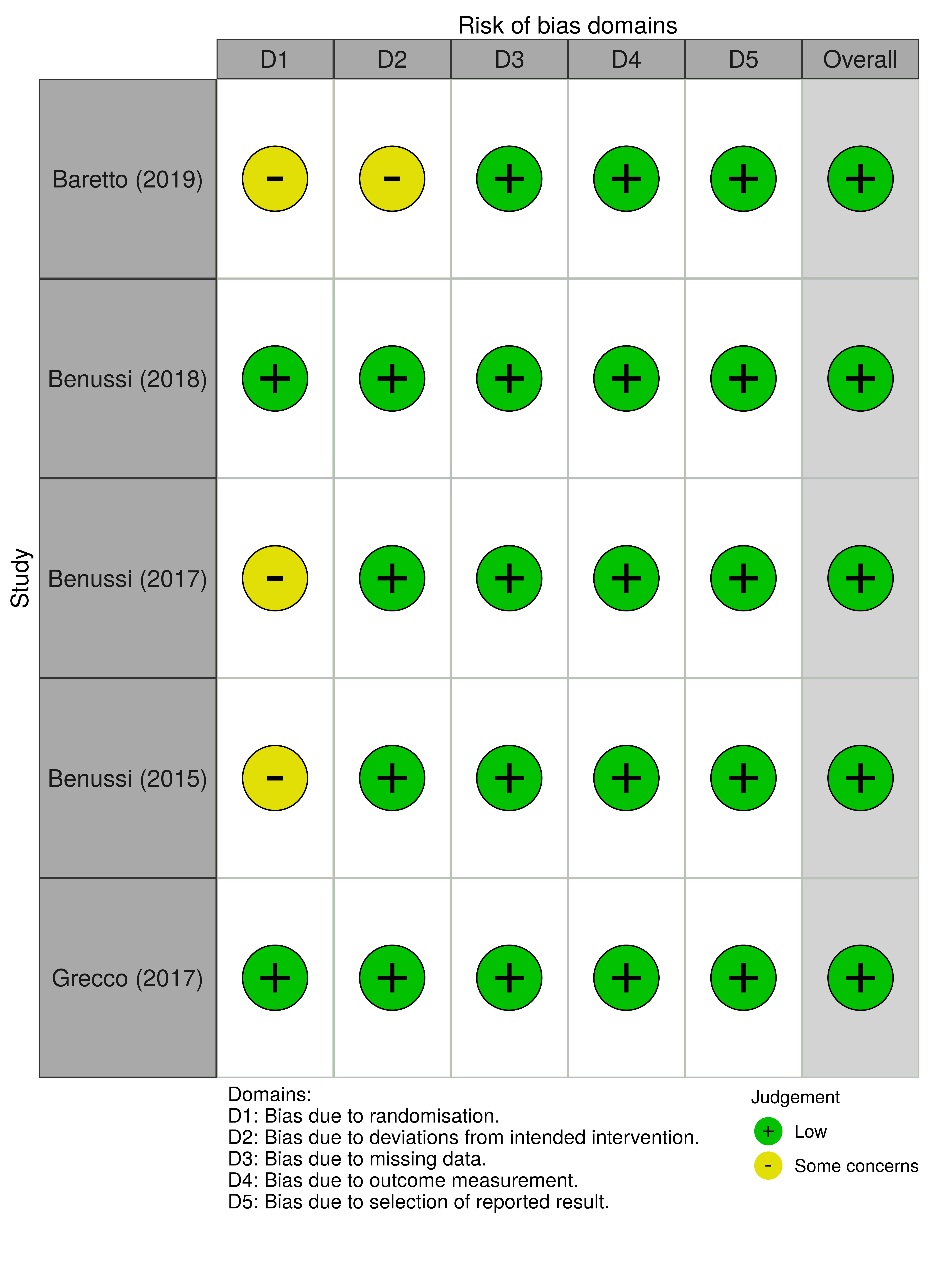

Supplement: 12311_2020_1181_MOESM3_ESM — Supplementary Fig.1 Assessment of the quality of included randomized controlled trials with the Cochrane Risk of Bias (RoB2) tool. The following domains of potential bias were considered: (D1) randomization process, (D2) deviations from the intended interventions, (D3) missing outcome data, (D4) measurement of the outcome, and (D5) selection of the reported result [file NIHMS1623197-supplement-12311_2020_1181_MOESM3_ESM.jpeg]
